# How Accurately Can Parents Judge Their Children’s Boredom in School?

**DOI:** 10.3389/fpsyg.2016.00770

**Published:** 2016-06-30

**Authors:** Ulrike E. Nett, Elena C. Daschmann, Thomas Goetz, Robert H. Stupnisky

**Affiliations:** ^1^University of UlmUlm, Germany; ^2^University of KonstanzKonstanz, Germany; ^3^Thurgau University of Teacher EducationKreuzlingen, Switzerland; ^4^University of North DakotaGrand Forks, ND, USA

**Keywords:** boredom, antecedents, coping, parents, students

## Abstract

The purpose of the present study was to explore what parents know about their Children’s boredom in school; specifically, the frequency, intensity, and antecedents of their Children’s boredom, as well as how they cope with boredom. A questionnaire was administered to 437 grade 9 students (54% female, *M*_age_ = 14.82) and their parents (72% mothers, 14% fathers, 12% both parents, *M*_age_ = 45.26) measuring variables related to students boredom in mathematics class. Three different measurements were used to evaluate the accuracy of parents’ judgments: (1) the correlation between parents’ and students’ answers, (2) the mean differences between parents’ and students’ answers, and (3) the mean values of absolute differences of parents’ and students’ answers. The results suggest that parents generally have an informed knowledge about their child’s boredom and related facets. This is reflected by a mean correlation of medium size (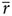 = 0.34) and a small mean effect size of the difference between parents’ and students’ judgments over all items (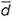 = 0.20). Parents are also substantially better in judging their Children’s boredom compared to guessing for all variables (mean effect size of 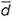 = 0.65). They had the most precise judgments for the frequency and intensity of boredom. The antecedents of boredom (e.g., characteristics of instruction) were also well estimated by parents; specifically, parents tend to have a bias in favor for their children evidenced by overestimating antecedents that cannot be influenced by the students and underestimating those that can be influenced by the students. The least concordance was found between parents’ and Children’s perception of boredom coping strategies (e.g., accepting boredom), implying that parents lack information about how their children intentionally cope with boredom. Implications for research on student boredom are discussed as well as practical applications involving parents in boredom prevention.

## Introduction

Boredom is a common phenomenon in modern society – even referred to by some as a plague (Spacks, 1995; Pekrun et al., 2010). Among children and adolescents in particular, boredom is often associated with school (Larson and Richards, 1991; Fallis and Opotow, 2003; Mora, 2011). Studies indicate that students experience this emotion during more than 30% of the time they spend in class (Larson and Richards, 1991; Nett et al., 2011).

Even though boredom is a prevalent emotion in students, it has so far received little attention from researchers (Vodanovich, 2003a; Pekrun et al., 2010). This might be due to boredom being accepted as a very common emotion experienced in school by students, thus teachers and parents regard it as a negative emotion that can be coped with. One aspect that is especially underexplored is the perceptions of students’ boredom by others such as teachers (except Daschmann et al., 2014) and parents.

During the past few decades parental involvement in schools has received increased attention due to its importance for both schools and families (Rogers et al., 2009; LaRocque et al., 2011; Cheung and Pomerantz, 2012; Jeynes, 2012). One way parents might be able to assist schools is by helping their children regulate of academic emotions, especially coping with boredom. Parents may make efforts to reduce their Children’s boredom (e.g., by fostering interest in certain subjects) and develop positive coping behaviors (e.g., by showing their children how they can reactivate attention). In order to support their children in coping with boredom, however, they need to validly and reliably perceive their Children’s boredom level, as well as the boredom related antecedents and coping strategies.

This lack of attention on boredom from differing perspectives (students, teachers, and parents) is troubling because students who are frequently experiencing boredom in class are at risk of several negative consequences, such as shallow information processing (Goetz and Hall, 2014), low attentiveness (Farmer and Sundberg, 1986), and less effort (Belton and Priyadharshini, 2007; Pekrun et al., 2010). These outcomes of boredom often lead to even more severe consequences including absenteeism (Hamilton et al., 1984) and drop out (Fallis and Opotow, 2003; Dube and Orpinas, 2009).

### Parents’ Perspectives on Students’ Boredom

If parents’ perceived their Children’s emotional experience fairly accurately, they might be able to support their children to find appropriate ways of coping with their emotional experiences. Parents can contribute valuable information to create more supportive and enriching learning environments for students because parent knowledge can transfer into teacher knowledge through, for instance, parent–teacher talks focusing on boredom.

Students are believed to be accurate judges of their own experiences of boredom, as well as the antecedents of boredom, because they know the situations that make them bored. Students’ perspectives on boredom and boredom related behavior is assumed to be based on authentic experiences of this emotion. Compared to other perspectives, such as parents, the students’ own perceptions of their boredom experience can be assumed to reflect these experiences quite validly (De Jong and Westerhof, 2001; Clausen, 2002; Kunter and Baumert, 2006; Lüdtke et al., 2006).

The parents’ perspective, however, is different than the students’ point of view. Parents do not have direct insight into the classroom and therefore they have to judge their Children’s emotional experience indirectly. Parents are informed by what their children tell them about their academic emotional experiences. Parents may also deduce their Children’s boredom based on their attitude, as well as actions and reactions they perceive in daily life at home and assume to be similar in the classroom. Currently no studies exist that explicitly examine students’ boredom from parents’ point of view. Some empirical studies exist, however, on parents’ judgment of their Children’s emotions (not including boredom), personality, stress, and behavior. Spooner et al. (2005) found low to moderate relationships between students’ ratings and their parents’ estimation on shyness. Also, Quartier and Rossier (2008) reported small correlations of parents’ judgments with their Children’s descriptions of personality factors, such as extraversion, responsibility, and emotional stability. Bagdi and Pfister (2006) reported parents underestimate their Children’s level of stress. Another study that found parents were overly optimistic concerning their Children’s behavior, revealing that parents tend to overlook some of their Children’s behavioral issues (Seiffge-Krenke and Kollmar, 1998). Bylund et al. (2005) similarly found parents judged their Children’s health behavior in a much more positive way than children described it themselves. Finally, Salbach-Andrae et al. (2008) and van der Meer et al. (2008) reported low to moderate accordance between parents’ perceptions and students’ statements on health related issues. In a meta-analysis, Achenbach et al. (1987) investigated studies providing scores from different informants for relatively specific emotional, behavioral, or temperamental problems in children and adolescents. They found only low correlations (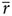 = 0.22) between participants themselves and other sources (e.g., parents or teachers). Taken together, these findings suggest parents tend to underestimate negative aspects of their Children’s emotional experience and overestimate aspects lying outside their Children’s control.

In order to investigate if parents can support their children in coping with boredom, a first step is to investigate how reliable parents are as sources of information regarding their Children’s boredom. Thus, the present study investigates how parents perceive their Children’s boredom in terms of frequency and intensity, the antecedents that lead to this emotion, and how their children cope with boredom.

### Students’ Experiences of Boredom

Only if parents judge the frequency and intensity of their childrens’ boredom accurately will they be able to address the problematic experience of boredom in school. It is important to note that boredom is not just a lack of interest; hence, boredom cannot be seen as the “opposite” of enjoyment or interest (Goetz and Hall, 2014), although the absence of interest may sometimes lead to boredom. Whereas boredom is emotionally distressing, lack of interest is affectively neutral and does not cause emotional distress (Goetz and Frenzel, 2006). When emotions are experienced in achievement settings, such as during classroom instruction or while doing homework, they can be referred to as academic emotions (Pekrun, 2006). While in some situations the experience of boredom has potential benefits, such as becoming more creative (e.g., Vodanovich, 2003a; Mann and Cadman, 2014), Tze et al. (2016) showed in a meta-analysis that, especially within academic settings, boredom is negatively related to many important academic outcomes and thus should be avoided.

Concerning the frequency of boredom, there is evidence showing boredom to be among the most commonly experienced emotions in academic settings (e.g., Robinson, 1975; Csikszentmihalyi and Larson, 1987). Daschmann et al. (2011) found 20% of ninth grade students to agree or strongly agree to “often feeling bored in mathematics class.” Results from experience sampling studies also reveal how often students experience boredom during classroom instruction. For example, Larson and Richards (1991) reported fifth and ninth grade students experience boredom during 32% of the time spent in class. An experience sampling study by Nett et al. (2011) showed that Grade 11 students claim some occurrence of boredom during 58% of classroom instruction. Similar results were found by Pekrun et al. (2010) among undergraduate university students who reported boredom was experienced in 42% of the assessed academic settings; thus, boredom was revealed as the most prevalent negative emotion.

Several studies examining boredom and other emotions in academic settings reveal boredom to be experienced quite strongly by students. For example, Skinner et al. (2008) reported fourth to seventh grade students with a mean value of 2.42 (possible range 1–4) for the intensity of their boredom experience. In university settings boredom has been found at substantial intensities by Pekrun et al. (2011), who reported a mean value of 30.84 (possible range 11–55) of boredom intensity experienced in class settings. Pekrun et al. (2010) also reported a mean value of 30.69 (possible range 11–55) of boredom intensity experienced among university students. Another important consideration is the intensity of boredom across academic subjects. The following studies all reveal mean levels for student boredom intensity near the scale midpoint across different subject domains: Collier (2011; *M* = 3.33 for Grade 8 students in mathematics; possible range 1–5), Goetz et al. (2006a; *M* = 2.80 for Grade 7–10 students in Physics; possible range 1–5), Goetz et al. (2007b; *M* = 2.86 for Grade 11 students in German class; possible range 1–5), and Goetz et al. (2006b; *M* = 2.83 for Grade 7–10 students in German lessons; possible range 1–5). In sum, these findings display a frequent and intense (close to or above the scale midpoint) occurrence of boredom among students of various age groups and across different academic domains. The question arises if parents are aware of the pervasiveness and intensity of boredom in school.

### Antecedents of Students’ Boredom

In order for parents to help their children feel less bored in school, it would be necessary for parents to accurately judge the antecedents’ (i.e., precursors or factors that influence occurrence) of their childrens’ boredom. Research has identified several antecedents of boredom in school. Pekrun’s (2006) control-value theory of emotions describes how boredom, among other academic emotions, originates from subjective perceptions of control over situations and appraisals of value. These control and value beliefs are assumed to be influenced by the person’s personality as well as social environment. Generally speaking, this means that boredom is thought to have individual (subjective) as well as environmental antecedents, and that the influence of these environmental causes is mediated by the person’s perception of the situation. Thus, Goetz and Hall (2014) identify three categories of antecedents of boredom: first, antecedents that lie within the environment (e.g., monotony of the instruction); second, those that lie within the individual itself (e.g., a proneness to be bored); and third, those that are elicited by a misfit between environment and individual (e.g., underchallenging or overchallenging learning environments).

Concerning antecedents of academic boredom that lie within the environment, instructional aspects in general were found to be responsible for boredom in students (e.g., Goetz, 2004). Lohrmann (2008) found students cited characteristics of instruction when asked for the antecedents of their boredom. Monotony was confirmed to be a precursor to boredom by Robinson (1975), Hill and Perkins (1985), Daschmann et al. (2011), as well as Fiske and Maddi (1961). Robinson (1975) postulated that monotonously instructed classes are the most common antecedents of boredom among students. As the style of instruction seems to be of high importance, teachers should be aware of the importance of modifying their instruction to engage their students and giving their students the possibility to contribute to the arrangement of the learning environment. This emphasizes the importance of student-centered instructional settings. Furthermore, students are bored more frequently in subject domains that are perceived as useless. Robinson (1975) additionally took external factors, such as the teacher, peers, and parents into account. Value appraisals (as described by Robinson, 1975 and Pekrun, 2006) represented by lack of meaning or not valuing school material were found as important precursors to boredom in several studies (cf. Fiske and Maddi, 1961; Morton-Williams and Finch, 1968; Robinson, 1975; Daschmann et al., 2011; Pekrun et al., 2011). Mitchell (1993) also found that meaningful learning material can prevent students from being bored.

Dispositional factors, such as boredom proneness as described in the second category by Goetz and Hall (2014), were also found to contribute to students’ experience of boredom (e.g., Farmer and Sundberg, 1986; Vodanovich and Kass, 1990; Larson and Richards, 1991). According to Vodanovich (2003b) the construct of boredom proneness provides a two-factor structure: external stimulation represents a person’s need for external animation and excitement, whereas internal stimulation represents a person’s ability to self-initiate interest. Culp (2006) found External stimulation to be negatively related to the personality traits honesty/humility, emotionality, and conscientiousness; alternatively, internal stimulation was positively related with the personality traits extraversion, conscientiousness, and openness to experience. These results showed that the students’ personality might also play an important role in their experiences of boredom. Parents typically know their Children’s personality quite well and thus might be able accurately gage their boredom from this important perspective.

Finally, concerning Goetz and Hall’s third category of antecedents of boredom, poor adjustment of learning material to students’ achievement levels was found to be a predictor of boredom among students by Daschmann et al. (2011). Lohrmann (2008) also found boredom to occur prevalently in situations where students were either over or under challenged. Titz (2001) furthermore detected both overly low and high competence cognitions to be associated with boredom in academic situations. Preckel et al. (2010) found that highly gifted students are not bored more frequently in regular classes than their classmates. Gifted students are bored for other reasons, although after a transition to high gifted classes they report boredom less often due to being underchallenged.

Daschmann et al. (2011) developed scales for assessing the various antecedents of boredom, namely monotony, lack of meaning, opportunity costs, lack of involvement, teacher dislike, and generalized boredom (tendency to be easily bored) being over-challenged, being under-challenged. In their study, these Precursors to Boredom Scales showed good internal validity as boredom was indeed due to different antecedents. Furthermore, the scales provided strong relationships with various characteristics of instruction as well as academic achievement (i.e., mathematics grades).

Considering the three categories of antecedents of boredom (Goetz and Hall, 2014) in comparison with previous findings concerning parents’ judgment as described above, parents might overestimate antecedents of boredom that lie within the environment while underestimating the influence of those that lie within their children.

### Students’ Coping with Boredom

Until now, little research literature exists on coping with boredom (Vodanovich, 2003b; Nett et al., 2010). The term “coping” is a concept predominantly applied to stress, specifically how people respond to stress (Skinner et al., 2003). Nett et al. (2010) proposed a theoretical framework that adapts a stress coping framework (Holahan et al., 2005) to the context of boredom in school; specifically, they differentiated between coping with boredom by cognitive and behavioral approach strategies versus cognitive and behavioral avoidance strategies. Nett et al. (2010) found that cognitive approach strategies, such as reactivating attention, might be most promising in coping with boredom. For example, students who make themselves aware of the importance of the current issue are less likely to be bored than students that use other boredom coping strategies such as influencing the instruction or distracting oneself. These results have been supported by further studies (cf. Nett et al., 2011; Tze et al., 2013). Goetz et al. (2007a) asked students to respond to open-ended questions on how they cope with boredom and identified a set of general strategies that correspond to the above mentioned framework by Nett et al. (2010) such as ‘reactivating attention’ (cognitive approach), ‘influencing the instruction’ (behavioral-approach), ‘relaxing’ (cognitive avoidance), ‘distracting oneself’ (behavioral avoidance). Parents’ understanding of their Children’s coping strategies, however, has yet to be studied in hopes of helping parents support their children in effective boredom coping.

### Study Objectives

The present study sought to explore what parents know about their Children’s boredom in school. This objective is important because boredom is a highly prevalent emotion experienced in school and students avoiding boredom entirely seems unlikely. Parents might be able to help their children find effective ways to cope with this omnipresent emotion. For parents to be able to do so, however, it is important to they have valid and reliable insight into their Children’s experiences of boredom, the antecedents of boredom, and how the children try to cope with it. Exploring parents’ ability to diagnose their Children’s boredom and boredom-related constructs is a first step in helping parents to assist their children with this issue. As there are many findings indicating that academic emotions are domain specific in nature (Goetz et al., 2006b, 2007a; Collier, 2011; Haag and Goetz, 2012), it is important to refer to one specific subject when asking students and parents about academic boredom. As boredom has a medium to high occurrence in mathematics classes, mathematics is a subject domain of high importance, and is one of the subjects with the highest number of lessons per week, we chose the subject of mathematics. We addressed the following research questions:

How accurately do parents perceive the frequency and intensity of boredom in their children?
Based on the results of related studies examining different perspectives on emotional or behavioral problems in children and adolescents (e.g., Achenbach et al., 1987), we expected parents to underestimate the prevalence of boredom in their children due to a parental bias.
How accurately do parents judge the antecedents of their Children’s boredom?
Also underexplored are the antecedents of students’ boredom, and from the parents’ perspectives there are no empirical studies. We therefore acted upon the same assumption that parents’ judgments will have an underling bias, leading us to hypothesize that parents’ will overestimate boredom antecedents lying outside their childrens’ control (e.g., characteristics of instruction or the teacher’s personality) while underestimating antecedents of boredom lying within their children (e.g., lack of interest, the student’s personality).
How accurately do parents judge their Children’s strategies for coping with boredom?
As there is neither much theoretical nor empirical evidence concerning parents’ perceptions of strategies for coping with boredom, this aspect of the study was exploratory and no hypothesis was formed.

## Materials and Methods

To investigate what parents know about their Children’s boredom in terms of frequency, intensity, antecedents, and coping strategies, we developed a questionnaire with parallel wording for students and their parents. The survey was administered to students who were asked to have their parents fill out the corresponding parent questionnaire.

### Participants and Procedure

Data was collected from 29 ninth grade classes in 14 German high schools. The student sample consisted of 437 students (54% female), with a mean age of 14.82 (*SD* = 0.50). Regarding the three-track system of German high schools, 29% of students attended Hauptschule (lowest or general school), 29% attended Realschule (intermediate or apprenticeship preparatory school), and 42% attended Gymnasium (highest or college preparatory school).

The parent sample consists of the corresponding 437 parents. Seventy-two percentage of the parent questionnaires were completed by mothers, 14% by fathers, and 12% by both of the parents (2% unknown). The participating parents were on average 45.26 (*SD* = 4.71) years old.

Data collection with the student sample took place in a classroom setting conducted by trained testing personnel. Students then were given the parent questionnaire and asked to have their parents answer all of the questions at home. All questionnaires were sealed in separate envelopes to ensure participant anonymity.

### Measures

#### Frequency and Intensity of Boredom

To address our first research question, two items on the frequency of boredom and one item on the intensity of boredom were formulated. The two items on the frequency of boredom both asked how often the students are generally bored in mathematics: the first item had a response format of 1 = *almost never* to 5 = *almost always*, and the second item asked for the percentage of time students felt bored during mathematics class. These items were followed by a question on the intensity of boredom with the response format 1 = *not intensely at all* to 5 = *very intensely*. The intercorrelations of the items on frequency and intensity of boredom are in Appendix A.

#### Antecedents of Boredom

The items on antecedents of boredom derive from the set of categories reported by Daschmann et al. (2011). For the current study, a survey was formulated consisting of eight items that addressed specific antecedents of boredom in mathematics class: (1) the subject domain, (2) characteristics of instruction, (3) the teacher’s personality, (4) the class/fellow students, (5) a lack of interest in the topic, (6) the student’s personality, (7) the content material being too easy, and (8) the content material being too difficult. Students and respective parents were posed the following statements “When I am [my son/daughter is] bored in mathematics class it is generally due to [REASON]” (1 = *strongly disagree* to 5 = *strongly agree*). The intercorrelations of the items on antecedents of boredom are depicted in Appendix A.

#### Coping with Boredom

To assess coping with boredom we utilized the set of categories reported by Goetz et al. (2007a). The first set of items asked for the general strategies students use when they feel bored in class, “What do you [does your son/daughter] do in mathematics class when you are [he/she is] bored? Please give your accordance [estimation] to each of the following statements.” The proposed strategies included (1) reactivate attention, (2) influence the instruction, (3) relax, and (4) distract oneself. Furthermore, (5) accept boredom, although this is not actually coping was included (1 = *strongly disagree* to 5 = *strongly agree*). The intercorrelations of the items on general strategies for coping with boredom are depicted in Appendix B.

### Data Analysis

In our sample, students were nested within classes. Intra-class correlations were higher for the student sample than for the parent sample. This is in line with previous assumptions as students of one class share a common perception of their classroom. For the student sample, intra-class correlations ranged from ICC = 0.01 for the antecedent of accepting/sustaining boredom to ICC = 0.18 for coping with boredom by playing, providing a median ICC = 0.05. For the parent sample, intra-class correlations ranged from ICC = 0.00 for the antecedent of accepting/sustaining boredom to ICC = 0.10 for the antecedent characteristics of instruction, with a median ICC = 0.02. The design effect, which is a function of the intra-class correlation and the average cluster size, was as high as DEFF = 3.5, indicating that the clustering in the data needs to be taken into account during estimation (Satorra and Muthen, 1995). Therefore, analyses were performed with Mplus (Muthén and Muthén, 1998–2007) taking the nested structure of the data into account. Variation across school sites was neglectable.

To analyze and interpret the quality of parents’ judgments’ of their Children’s boredom we accounted for three different indicators: (1) the correlation between parents’ and students’ answers, (2) the difference between parents’ and students’ answers, and (3) the mean value of the absolute difference between parents’ and students’ answers. Each of these indicators provides important information and only a combined interpretation of these three indicators gives a comprehensive picture of parents’ ability to judge their Children’s boredom.

#### Correlation between Parents’ and Students’ Answers (1)

For each of the items, the correlation between parents’ and students’ answers was calculated to determine the strength of the relationship. The correlation can be interpreted as how parents would judge their own child in relation to other children. According to Cohen (1988) correlations coefficients are considered to represent weak (*r* ≥ 0.10), medium (*r* ≥ 0.30), or strong (*r* ≥ 0.50) relationships.

#### Differences of Parents’ and Students’ Answers (2)

Further calculations involved the differences between parents’ and students’ responses (parents minus students). For each item the percentage of parents who correctly judged their Children’s answer to an item (difference value of zero), as well as the percentages of parents who under- or overestimated their Children’s value (difference values between 1 and 4 scores) is shown in **Tables 1**–**3**. Additionally, mean values of items were compared between the student and parent sample, and a WALD-test of parameter constraints was performed to gain information about whether there is a general tendency of under- or overestimation concerning each specific item. To be able to interpret the degree of under- or overestimation, we refer to Cohen’s (1988) effect size considering effects as small (*d* ≥ 0.2), medium (*d* ≥ 0.5) or large (*d* ≥ 0.8).

**Table 1:** Characteristic values for frequency and intensity of boredom.

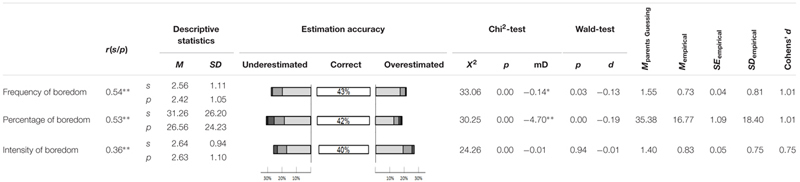

*^∗^p < 0.05; ^∗∗^p < 0.01; ^∗∗∗^p < 0.001. Depicted are correlations between students and parents answers, descriptive statistics, and results from Wald-tests of mean differences as well as Mean values of absolute differences of parents’ and students’ answers in relation to the mean expectancy values if parents were guessing their childrens’ answers. Response format ranged from 1 to 5 for intensity of boredom and frequency of boredom. Response format for percentage of boredom ranged from 0 to 100%. s: students’ values; p: parents’ values. In the schematic representation of the distribution of the degree of under- an overestimation of students’ reports on boredom by parents is depicted the percentage of parents who correctly judged their Children’s value on this item, as well as the percentages of parents who under- or overestimated their Children’s value by 1 = 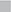, 2 = 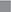, 3 = 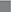, 4 = 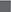 scores. The value M_parents Guessing_ was calculated by identifying the expectancy value of the absolute difference between parents’ and students’ judgments for each parent–student dyad hypothesizing that parents were only guessing by a given students’ answer. For the response format 1–5, the expectancy value of the absolute difference between parents’ and students’ judgments e given the students answer s was as follows: e = 2 if s = 1; e = 1.4 if s = 2; e = 1.2 if s = 3; e = 1.4 if s = 4; e = 2 if s = 5. For the percentage of boredom scale with the value of students answer s ∊ [0,100], the expectancy value of the absolute difference between parents’ and students’ judgments e was calculated as follows: 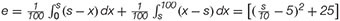. Finally, the mean score of all expectancy values was calculated. The value of Cohens’ d reflects the effect size of the difference between the values M_parents Guessing_ and M_empirical_ (reflecting the mean of the absolute difference between parents’ and students’ answer. In order to calculate Cohens’ d it was assumed that both samples provide the identical standard deviation which was empirically found in the sample (Cohen, 1988).*

**Table 2:** Characteristic values for antecedents of boredom.

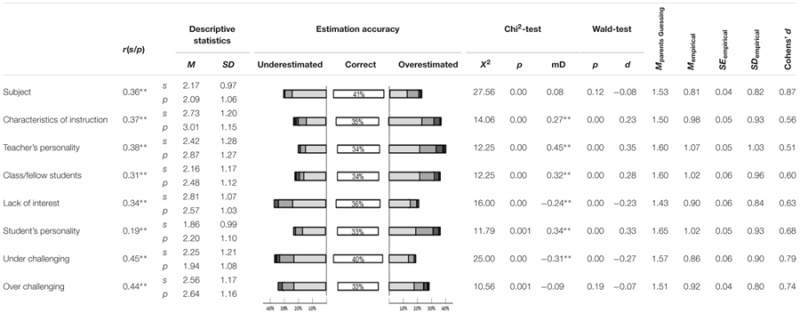

*^∗^p < 0.05; ^∗∗^p < 0.01; ^∗∗∗^p < 0.001. Depicted are correlations between students and parents answers, descriptive statistics, and results from Wald-tests of mean differences as well as Mean values of absolute differences of parents’ and students’ answers in relation to the mean expectancy values if parents were guessing their childrens’ answers. Response format ranged from 1 = Strongly disagree to 5 = Strongly agree. s: students’ values; p: parents’ values. In the schematic representation of the distribution of the degree of under- an overestimation of students’ reports on boredom by parents is depicted the percentage of parents who correctly judged their Children’s value on this item, as well as the percentages of parents who under- or overestimated their Children’s value by 1 = 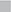, 2 = 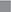, 3 = 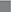, 4 = 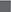 scores. The value M_parents Guessing_ was calculated by identifying the expectancy value of the absolute difference between parents’ and students’ judgments for each parent–student dyad hypothesizing that parents were only guessing by a given students’ answer. For the response format 1–5, the expectancy value of the absolute difference between parents’ and students’ judgments e given the students answer s was as follows: e = 2 if s = 1; e = 1.4 if s = 2; e = 1.2 if s = 3; e = 1.4 if s = 4; e = 2 if s = 5. Finally, the mean score of all expectancy values was calculated. The value of Cohens’ d reflects the effect size of the difference between the values M_parents Guessing_ and M_empirical_ (reflecting the mean of the absolute difference between parents’ and students’ answer. In order to calculate Cohens’ d it was assumed that both samples provide the identical standard deviation which was empirically found in the sample (Cohen, 1988).*

**Table 3:** Characteristic values for coping with boredom.

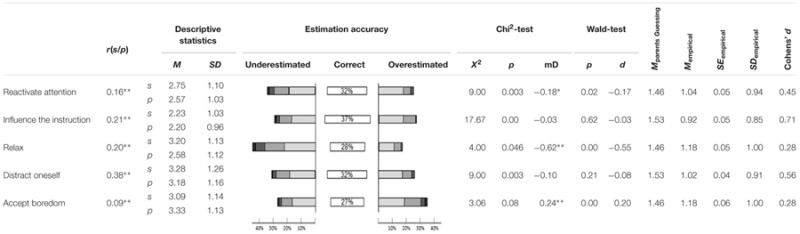

*^∗^p < 0.05; ^∗∗^p < 0.01; ^∗∗∗^p < 0.001. Depicted are correlations between students and parents answers, descriptive statistics, and results from Wald-tests of mean differences as well as Mean values of absolute differences of parents’ and students’ answers in relation to the mean expectancy values if parents were guessing their childrens’ answers. Response format ranged from 1 = Strongly disagree to 5 = Strongly agree. s: students’ values; p: parents’ values. In the schematic representation of the distribution of the degree of under- an overestimation of students’ reports on boredom by parents is depicted the percentage of parents who correctly judged their Children’s value on this item, as well as the percentages of parents who under- or overestimated their Children’s value by 1 = 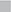, 2 = 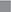, 3 = 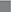, 4 = 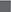 scores. The value M_parents Guessing_ was calculated by identifying the expectancy value of the absolute difference between parents’ and students’ judgments for each parent–student dyad hypothesizing that parents were only guessing by a given students’ answer. For the response format 1–5, the expectancy value of the absolute difference between parents’ and students’ judgments e given the students answer s was as follows: e = 2 if s = 1; e = 1.4 if s = 2; e = 1.2 if s = 3; e = 1.4 if s = 4; e = 2 if s = 5. Finally, the mean score of all expectancy values was calculated. The value of Cohens’ d reflects the effect size of the difference between the values M_parents Guessing_ and M_empirical_ (reflecting the mean of the absolute difference between parents’ and students’ answer. In order to calculate Cohens’ d it was assumed that both samples provide the identical standard deviation which was empirically found in the sample (Cohen, 1988).*

#### Mean Values of Absolute Differences of Parents’ and Students’ Answers (3)

We also calculated the mean, standard deviation, and standard error of the absolute value of parents’ judgment differing from their own Children’s statements. This indicator shows the degree to which parents are able to estimate their Children’s appraisals and behavior correctly. A mean value of zero would occur if all parents judge their Children’s statements correctly, the more the mean value differs from zero, the more the parents misjudge their children’ s statements. To be able to interpret the degree of misjudgment, the expectancy value of parents blindly guessing their Children’s answers was calculated for each parent–student dyad. This was calculated using the expectancy value of the absolute difference of parents’ guessed answer and the students answer. For a description of this calculation in detail, see Appendix C. The mean expectancy value of parents guessing their Children’s answers was also calculated (cf. notes of **Tables 1**–**3**)

The value of the concordance between parents and Children’s answers can be interpreted in relation to two hypothetical events: first, in relation to the event of parents knowing or having some knowledge of their Children’s answers; and second, the event parents guessing their Children’s answers (results presented in **Tables 1**–**3**). To be able to interpret the dimension of the difference between parents’ empirical value and the value of parents guessing, an effect size according to Cohen’s *d* (Cohen, 1988, interpretation see above) was calculated. Therefore, we assumed the two samples, the empirical sample and the hypothesized sample of parents’ guessing, to have the same standard deviation (cf. Cohen, 1988).

#### Combined Interpretation

When comparing the two different perceptions of the same construct by parents and students, it is important to concurrently regard the three indicators of (1) the correlation, (2) the difference, and (3) mean values of absolute difference of parents and students answers. Only the combination of all three analyses allows for specific interpretation. For example, items providing a high correlation corresponding with a low difference in mean values would be related to a low mean value of absolute difference and can be interpreted to reflect a high convergence between students’ and parents’ answers. This would lead to the conclusion that parents have a good judgment of their children in that area. The case of an item resulting in a high correlation and high difference in mean score would lead to the interpretation that parents can judge their own Children’s boredom in relation to other children quite well, but that parents in general overestimate (or underestimate) the level of boredom in children. In this case, also the mean value of the absolute difference would be relatively high. The pattern of an item resulting in a low correlation and low difference in mean value would occur when the parent sample and the student sample provide a similar mean score, but when parents are not able to estimate their Children’s answers in relation to classmates and overestimate or underestimate their own Children’s value unsystematically. In this case, the mean value of the absolute difference provides information about the normative accuracy of the parents’ estimation. Alternatively, an item providing a low correlation and a large mean score difference would also provide a large mean value of absolute difference and reflect an imprecise perception parents have of their children in that area.

### Further Data Analysis

For further interpretation of our data, we calculated average values of the correlations (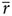) and effect sizes (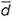) among each set of items. For averaging the correlations, we performed a Fisher-z standardization of each correlation, calculated the mean value of these standardized correlations, and transferred them back to unstandardized correlations. For averaging the effect size of each set of items, we calculated a mean value of the absolute values of each effect size in that set of items.

## Results

### Frequency and Intensity of Boredom

**Table 1** shows the results for the items on frequency and intensity of boredom, comparing parents’ answers to their Children’s responses. These items revealed a medium to high (cf. Cohen, 1988) correlation with a mean score of 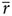 = 0.48. On average, 41.7% of the parents correctly judged their child’s scores for items on frequency and intensity of boredom. When taking into account those parents who over- or underestimated their children by ±1 score, the percentage is even higher at 81.7%. The mean effect size of the differences of parents’ and students’ answers is 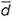 = -0.11, which is very low (cf. Cohen, 1988) and all effect sizes were negative; thus, parents tended to slightly underestimate their childrens’ boredom.

**Table 1** shows further the mean, standard deviation, and standard error of the absolute difference between students’ and parents’ response. The interval of the possible difference between students’ and parents’ response ranges from zero (parents know their Children’s answer) to the expectancy value of parents guessing their Children’s answer. Generally, the mean absolute difference between students’ and parents’ estimation on frequency and intensity of boredom were significantly below the expectancy value of the probability of parents just guessing their Children’s statements. With a large mean effect size of 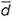 = 0.93, it was concluded that parents’ judgments of their own child’s answers are far better than chance. That is to say, parents generally perceive the intensity and frequency of the Children’s boredom quite well. The lower correlation of the items on intensity of boredom, however, is indicating that parents might not be able to judge the intensity of boredom of their children in relation to their classmates as well as the frequency. Their judgments might still be considered quite accurate keeping in mind that parents have no insight into their Children’s’ classmates’ experiences of boredom

### Antecedents of Boredom

**Table 2** shows the antecedents of boredom item analysis results. A mean correlation of 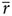 = 0.36 reveals a medium relationship (Cohen, 1988) between students and their parents. On average, 35.8% of parents made accurate estimations for their Children’s antecedents of boredom, and 78.7% of parents’ judgment only differed of maximum ±1 score from their Children’s answer on these items. The mean effect size of the Wald-test comparing parents’ and students’ answers was 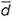 = 0.23, which means that parents’ judgments differed at some points meaningful from students’ reports. Four antecedents were significantly overestimated by parents: characteristics of instruction, teacher’s personality, the class, and students’ personality. Two antecedents of boredom were significantly underestimated by parents: lack of interest and underchallenged students. For the reasons of boredom due to “overchallenging” and “the subject,” parents neither tended to over- nor underestimate their Children’s answers.

The mean of the absolute difference between students’ and parents’ estimations of the antecedents of boredom items (**Table 1**) was *M* = 0.95. All mean absolute differences are significantly below the expectancy value of parents guessing, with a medium mean effect size of 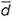 = 0.67. The results show that parents are fairly good predictors of the antecedents of their Children’s boredom; however, the findings are not in line with the hypothesis that the students’ personality as an antecedent of boredom is overestimated rather than underestimated by their parents.

### Coping with Boredom

The results regarding students’ coping strategies and their parents’ estimations can be found in **Table 3**. The correlations of parents’ estimations with students’ answers revealed a rather small mean correlation of 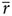 = 0.21. Overall, only 31.2% of parents correctly judged their Children’s answers, and 69.6% of parents made nearly correct judgments (±1 score difference). Results of this set of variables provided a low mean effect size of 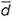 = 0.21. There was no tendency of under- or overestimation found regarding the strategies of reactivating attention, making an impact on the instruction, or distracting oneself, which lead to no effect for the differences in mean scores. The strategy of relaxing was significantly underestimated by parents, while accepting/sustaining boredom was slightly overestimated.

Regarding the mean absolute difference (**Table 3**) of *M* = 1.07, with only a small mean effect size (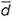 = 0.46) parents were not just guessing their students’ answers.

### Summary

Taking all items into account, our data leads to a mean correlation of medium size (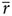 = 0.34). Further, items overestimated by parents provided a mean effect size of 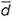 = 0.28, whereas the mean effect size of all items being underestimated by parents came to 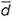 = -0.16. This shows that the overall degree of overestimation was slightly higher than underestimation, but still very low. Generally, the mean effect size (not considering the algebraic sign) concerning the difference between parents’ and students’ judgments over all items was 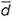 = 0.20, which represents only a slight asymmetry between parents’ and students’ perceptions. With a mean effect size of 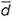 = 0.65, this reflects parents as substantially better at judging their Children’s boredom than guessing for all variables.

## Discussion

The goal of the present study was to investigate what parents know about their Children’s boredom. The results revealed that parents generally have accurate insight into their Children’s boredom as well as into possible antecedents and coping strategies. In particular, parents are good judges of how intense and how often their children are bored in mathematics class. Parents also fairly reliably perceive their Children’s antecedents, although they tend to have a bias in favor of identifying antecedents that are not controllable by their children. Parents furthermore perceive general coping strategies quite well, although they tend to underestimate some coping strategies students’ can actively engage in to reduce boredom.

### Frequency and Intensity of Boredom

As theory implies (cf. above, e.g., Bylund et al., 2005; Salbach-Andrae et al., 2008; van der Meer et al., 2008), parents slightly underestimate their Children’s boredom. Also in line with previous results (Larson and Richards, 1991; Nett et al., 2011), however, both parents and their children claim that students are bored almost a third of the time they spend in class. This reflects a dissipation of human resources that achievement oriented societies cannot afford, and confirms the urgency to help students by preventing boredom. In more detail, it also reflects that parents seem to be aware of the occurrence of this problematic emotion in school settings. In general, parents seem to have accurate judgments of how often their children are bored during mathematics class. This knowledge of parents might be a potential to support students in addressing their emotion in an adequate way.

### Antecedents of Boredom

Parents are aware of the frequency and intensity of their Children’s boredom, but if they also have insight into the causes of their Children’s boredom they might be able to support their children to cope adequately with it. In general, parents seem to know quite well what causes boredom in their own child in relation to other children, as shown by generally medium correlations Specific antecedents of boredom, however, are systematically over- or underestimated by parents. This might have consequences on how parents help their children deal with this emotion.

Some external antecedents (Goetz and Hall, 2014) in particular are not experienced by students as antecedents of boredom as much as parents assume them to be. The antecedents “characteristics of instruction,” “teacher’s personality,” and “fellow students” (Daschmann et al., 2011) provide on the one hand medium correlations, indicating that they are estimated quite accurately by parents for their own child in relations to others. On the other hand, these antecedents were systematically overestimated by the parent sample. A reason for this could be that, as hypothesized, parents’ answers might be biased in terms of trying to search for external explanations for their Children’s boredom, such as blaming others including the teacher or classmates. Alternatively, it is also in line with our hypothesis that “lack of interest” as an antecedent of boredom that lies within the individual (Goetz and Hall, 2014) is underestimated by parents. In total, these findings are in line with the previous studies presented above (cf. Seiffge-Krenke and Kollmar, 1998; Bylund et al., 2005; Salbach-Andrae et al., 2008; van der Meer et al., 2008). However, not in line with our hypothesis is that the students’ personality is another antecedent of boredom overestimated by parents.

Further, it is remarkable that the items over- and underchallenging students provide the highest correlations between students’ answers and parents’ estimations. Parents seem to know quite well if their child (in relation to others) is over or under challenged in mathematics class and if their boredom is due to this reason. Yet in general, parents underestimate the occurrence of underchallenging students in mathematics. These results might reflect that boredom, as a negative but ‘silent’ emotion, is generally accepted by both students’ and parents to be often experienced in school. Boredom seems to be perceived as an emotion that cannot be prevented, thus antecedents that can be influenced by the students themselves (e.g., interest) might be underestimated by parents. In contrast, antecedents that cannot be influenced by the students but have to be accepted (e.g., teacher’s personality) might be overestimated (cf., Bylund et al., 2005).

### Coping with Boredom

The results for boredom coping strategies were quite different from the antecedents of boredom. For coping with boredom in general, the relationship between students’ answers and parents’ estimations resulted in lower correlations. This shows that parents do not know what strategies their own child generally uses when coping with boredom in class. Although the low effect sizes concerning the mean differences reveals that parents are still aware of the kinds of strategies students generally use to cope with boredom. One exception was that the strategy of relaxing is used by students much more often than parents would think. This might be due to the fact that the experience of boredom is not as negative for students as parents assume it to be. Students might even enjoy to some degree the possibility to relax their mind. Also, parents underestimate the use of the strategy of reactivating attention, the strategy that previous literature found to be most adequate in order to cope with boredom (e.g., Nett et al., 2011; Tze et al., 2013). These results reflect that parents’ might not talk to their children about intentional strategies to cope with boredom. This might be due to the fact that they either accept their Children’s experience of boredom or due to the fact that they are not aware that their children are able to use intentional strategies to cope effectively with their boredom.

## Conclusion

Overall, it is remarkable how precisely parents can judge the experience of their Children’s boredom in various facets. Correlations between students’ and parents answers yielded mostly medium or high effect sizes, thus suggesting that parents’ can interpret their child’s experience of facts of boredom in relation to the fellow students rather well.

When looking at the percentage of parents who correctly estimated their Children’s answer to the items it becomes obvious that around 30% neither over- nor underestimated their Children’s boredom (range: 27–43%; cf. **Tables 1**–**3**). However, in line with previous studies that compared parents’ judgment to students’ answers (cf. Bylund et al., 2005), parents seem to be overly optimistic regarding their Children’s experience of boredom and have a bias in favor of their children. Also, the average effect size of parents’ judgments in relation to the expectancy value of guessing was remarkably large, especially given the fact that parents are not in the classroom with their children but have to infer their experience of boredom and their reaction to this emotion indirectly.

### Limitations

The present study is the first investigation of parents’ perspectives on their Children’s experiences of boredom during school instruction. Exploratory research objectives give first insight, but in order to interpret and draw well founded conclusions of the results a global theory on how parents can judge students boredom would be beneficial. For a deeper understanding and possible interpretations on the basis of our results, a broader theoretical approach would have been optimal.

Furthermore, due to the domain specificity of boredom, we only focused on mathematics. It is also possible that the researched aspects of boredom might be differently perceived by students and parents in other subject domains. Future research should therefore also take other school subjects into account. We do not have a hypothesis why parents’ diagnostic abilities should differ across subjects. Although, it might be the case that parents and their children talk more about subjects of high importance at home, thus, parents’ might be better informed about their Children’s experience of boredom within subjects of high value or a high number of lessons per week.

This study was also conducted solely on German students and their parents. It would be beneficial to expand the student and parent samples to other countries and cultures to capture the huge variety of different school systems and diverging parent involvement. For an even broader view on boredom in students, the teachers’ perspective could be integrated in a future study design to enhance the overall picture of the constructs.

Another aspect that should be taken into account for future study designs would be to integrate scales for the specific constructs instead of single-item measures, and to integrate the experience sampling method in order to assess state experiences. It would be possible, for instance, to expand the measurement of students’ perceptions by integrating state measurements of their experience of boredom in terms of frequency and intensity, as well as actual antecedents and how students cope with this emotion during classroom instruction. For the parent sample state measurements would not be possible; however, it may be the case that parents’ estimations are found to be more accurate if assessments of students’ boredom are more rigorous.

### Implications

Parents seem to know how much their children experience boredom, further they seem to be quite well aware of the antecedents of this emotion. However, parents might not have enough information about their children’ use of coping strategies, such as intentionally avoiding factors that lead to this emotion and helping them develop more efficient coping strategies such as reappraisal. Therefore, the current study supports future research on promoting helping strategies for parents to reduce boredom in their children in school. One way of integrating parents’ knowledge about their Children’s boredom could include more parent involvement in school. For example, by giving parents the opportunity to share ideas with teachers and develop strategies together in order to prevent boredom for their own child, but also strategies which could be applicable more generally. Therefore, when developing and implementing intervention programs, it would be necessary to integrate parent components that support parents in practicing more efficient coping strategies with their children.

On a broader scale, parents should be involved in more educational research to share their specific knowledge about children and understand the different perspectives on school. For teachers and researchers it is also important to be informed about the preciseness of parents’ perception of students’ boredom. When involving parents in boredom reducing actions, they should be aware of the specific perception parents have of their Children’s boredom.

## Author Contributions

UN and ED drafted the work which was revised critically by TG and RS. UN, ED, and TG contributed to all steps of the work. RS contributed to the Interpretation of the data for the work. All authors approve of the final version of the manuscript and agree to be accountable for all aspects of the work in ensuring that questions related to the accuracy or integrity of any part of the work are appropriately investigated and resolved.

## Conflict of Interest Statement

The authors declare that the research was conducted in the absence of any commercial or financial relationships that could be construed as a potential conflict of interest.
