# Supplementary material for: How Accurately Can Parents Judge Their Children’s Boredom in School?
Source: Front Psychol. 2016 Jun 30;7:770. doi: 10.3389/fpsyg.2016.00770 (PMC4927813; doi:10.3389/fpsyg.2016.00770)
Supplement: Supplementary file 1 [file Data_Sheet_1.PDF]

Appendix A

Intercorrelations of Antecedents of Boredom.

	1	2	3	4	5	6	7	8	9	10	11
1. Frequency of Boredom	--	.79**	.66**	0.42**	.34**	.38**	-.02	.31**	.15**	-.09	.35**
2. Frequency of B. (Percentage)	.77**	--	.61**	.39**	.34**	.37**	-.03	.26**	.15**	-.09	.33**
3. Intensity of Boredom	.62**	.57**	--	.31**	.29**	.33**	-.02	.26**	.15**	-.14**	.31**
4. Subject	.42**	.22**	.25**	--	.25**	.19**	-.07	.39**	.22**	-.33**	.35**
5. Characteristics of instruction	.28**	.22**	.25**	.09*	--	.66**	.05	.20**	.18**	-.11*	.15**
6. Teacher's personality	.28**	.22**	.29**	.13**	.67**	--	.14**	.23**	.23**	-.07	.12*
7. Class / fellow students	.01	-.07	.12*	.00	.20**	.28**	--	.05	.17	.04	-.03
8. Lack of interest	.29**	.22**	.23**	.54**	.12*	.07	.04	--	.22**	-.15**	.29**
9. Student's personality	.22**	.14**	.17**	.39**	.07	.20**	.20**	.38**	--	-.07	.14**
10. Under challenging	-.11*	-.09	-.03	-.15*	-.03	.00	.11*	-.07	-.00	--	-.43**
11. Over challenging	.25**	.18**	.42**	.42**	.20**	.23**	.06	.37**	.27**	-.10	--

Note. Depicted are intercorrelations of items on frequency and intensity of boredom as well as on antecedents of boredom. The students' intercorrelations are above the diagonal, the parents' below the diagonal.

Appendix B

Intercorrelations of General Coping with Boredom.

	1	2	3	4	5
1. Reactivate attention	--	.10	.11*	-.05	.02
2. Influence the instruction	.26**	--	-.03	-.05	.00
3. Relax	.11*	.01	--	.43**	.25**
4. Distract oneself	.07	-.05	.33**	--	.03
5. Accept boredom	-.11*	-.25**	.15**	.23**	--

Note. Depicted are intercorrelations of items on general strategies for coping with boredom. The students' intercorrelations are above the diagonal, the parents' below the diagonal.

Appendix C

In general, the expectancy value e_s of the parent's judgment (i) differing from the student's judgment s on an n-point Likert scale ranging from 1 to n is calculated using the following formula:

$$e_s = \frac{\sum_{i=1}^{n} |i - s|}{n}$$

To make this point more clear, we have two examples:

(1) Suppose a student answered a question with the value 1 on a 5-point Likert scale. If a parent was just guessing the parent's answer is 1, 2, 3, 4 or 5, all possible answers have the same probability (1/5). The expectancy value e of the parent's judgement differing from the student's judgement can be calculated the following way:

$$e = \frac{\sum_{i=1}^{5} |i - 1|}{5} = \frac{|1-1| + |2-1| + |3-1| + |4-1| + |5-1|}{5} = \frac{0+1+2+3+4}{5} = 2$$

(2) If a student answered a question with the value 3 on a 5-point Likert scale, the respective expectancy value of the absolute difference is:

$$e = \frac{\sum_{i=1}^{5} |i - 3|}{5} = \frac{|1-3| + |2-3| + |3-3| + |4-3| + |5-3|}{5} = \frac{2+1+0+1+2}{5} = 1.2$$

For the percentage of boredom scale with the value of students answer $s \in [0, 100]$, the expectancy value of the absolute difference between parents' and students' judgments e was calculated as follows: $e = \frac{1}{100}\int_0^s (s - x)dx + \frac{1}{100}\int_s^{100} (x - s)dx = \left[(\frac{s}{10} - 5)^2 + 25\right]$
